# Development of a Dual Photoacoustic–Ultrasound Imaging System: Current Status and Future Perspectives

**DOI:** 10.3390/s26030823

**Published:** 2026-01-26

**Authors:** Van Hiep Pham, Tuan Nguyen Van

**Affiliations:** 1 Faculty of Mechanical Engineering and Mechatronics, PHENIKAA School of Engineering, PHENIKAA University, Nguyen Trac, Duong Noi, Hanoi 12116, Vietnam; tuan.nguyenvan@phenikaa-uni.edu.vn; 2 PHENIKAA Research and Technology Institute (PRATI), A&A Green Phoenix Group JSC, No. 167 Hoang Ngan, Yen Hoa, Hanoi 11313, Vietnam

**Keywords:** dual photoacoustic and ultrasound imaging, PAUS probe design, PAUS system design, photoacoustic imaging, ultrasound imaging

## Abstract

Integrated photoacoustic and ultrasound (PAUS) imaging is a promising technology for both preclinical and clinical applications, as it exploits both advantages of photoacoustic (PA) and ultrasound (US) imaging in high resolutions and acoustic penetration depth, respectively. Using a shared US transducer, data acquisition (DAQ), and signal processing framework, the PAUS system provides simultaneous functional and anatomical information. To date, numerous studies have been reported to demonstrate the capabilities and proposed innovative approaches for the development of the PAUS probes and systems. Key performance parameters, including probe resolution, extending the region of interest (ROI), and increasing the scanning speed, play critical roles in improving image quality, expanding the scanning area, and reducing the scanning time, respectively. This review aims to summarize recent advances in PAUS probes and systems designed for rapid image acquisition. The principles and signal processing are introduced as the fundamentals for designing the PAUS probes and systems. The summaries of the PAUS probe and system design are presented and compared systematically. Furthermore, new approaches in the development of PAUS probes and systems are proposed to enhance their proficiencies in preclinical and clinical applications.

## 1. Introduction

PA imaging is an emerging, non-invasive technology capable of visualizing molecular functional information of tissue [1,2,3,4,5,6]. When pulsed laser light illuminates tissue, optical absorption induces thermoelastic expansion, generating ultrasound (US) waves that are subsequently detected by US transducers to reconstruct high-resolution PA images. According to the PA principles, this modality has enabled numerous applications ranging from preclinical animal [7,8,9,10,11,12] to clinical human studies [13,14,15,16,17,18]. However, PA relies on optical absorption in tissue, which limits its ability to provide structural information.

US imaging techniques have been widely adopted in clinical applications due to their capability in non-invasive, deep penetration, high spatial resolution, and real-time imaging [19,20]. Based on the different mechanical characteristics of tissue, US imaging visualizes the general structural information that is usually not visible in PA imaging [21,22]. One of the main drawbacks of US imaging is its difficulty in distinguishing tissues that have similar acoustic properties, thereby leading to diagnostic inaccuracies [23].

Using the same US transducer and DAQ, the PAUS technique synergistically combines the advantages of both PA and US imaging modalities [24,25]. Consequently, PAUS imaging offers concurrent functional and anatomical information by acquiring PA and US signals simultaneously at each lateral resolution [26]. Several studies have been published, which show the PAUS’s effectiveness in preclinical animal applications [23,27,28,29,30,31,32,33,34,35,36,37,38,39,40,41]. Utilizing the PAUS technique, accurate data are obtained, which are used for clinical diagnostic and therapy-monitoring purposes [42]. Extensive research has highlighted the broad clinical potential of PAUS imaging, and these applications have been comprehensively reviewed [43,44]. They are categorized into some groups, including healthy tissue [45,46,47], tumor and metastasis [48,49,50,51,52,53], bones and joints [54,55], cardiovascular [56,57,58,59], thyroid [60], breast [61,62,63], skin [64,65], extremities [66], large blood vessels [67], prostate [68,69], placenta [70,71], bowels [72,73,74], periodontal health [75], and spine in human cadavers [76].

Despite these promising applications, developing PAUS systems remains challenging for both research and commercial deployment. Several commercial PAUS systems have been used for preclinical purposes, such as the iU22 Philips Healthcare [77], VevoLAZR series—FujiFilm VisualSonics [78], MSOT Acuity series—iThera Medical [79], and Vantage series—Verasonics [69]. These systems enable PAUS imaging without the need for additional hardware or software modifications. However, their widespread adoption is limited by high system costs.

In academic research, PAUS system development has focused on improving image quality, extending the ROI, and reducing scanning time. Image quality depends on the spatial resolution of the PAUS probe, which is optimized through the integration of optical and acoustic components. Probes are typically designed using single elements, linear arrays, or customized US transducers. In addition, advanced image processing algorithms are required to further enhance image quality. Accordingly, this review categorizes PAUS probe designs based on single-focused elements, linear arrays, and customized transducers.

To extend the ROI and reduce scanning time, the PAUS system was developed by exploiting the motions of some mechanisms. The PAUS probe is attached to the movement part of the PAUS system, which is controlled to perform the motion in one-dimensional (1D) or two-dimensional (2D) directions. Linear array-based PAUS systems can achieve B-scan imaging without mechanical motion, whereas systems employing single focused element transducers require 1D scanning. Consequently, 3D imaging is obtained through 1D motion for linear arrays and 2D motion for single-element transducers. The ROI can be expanded by increasing the scanning range, while scanning time is minimized by maximizing the scanning speed. Technically, the scanning speed is determined by the pulse repetition rate (PRR) of the laser function generator, pulser/receiver (P/R), and motion speed of the PAUS system. In that case, PAUS systems are typically designed to operate at the highest feasible scanning speed.

This review will focus on the current development of PAUS probes and systems that can provide an acceptable image quality with a suitable ROI in a short time. These specifications need to be optimized for diagnostic decision-making. The PAUS principle and signal processing are presented, which are used to address the design technologies of PAUS probes and systems. These design technologies are shown comprehensively and summarized systematically. The pros and cons of each design version are discussed. Based on the discussion, future work for developing PAUS probes and systems is proposed to enhance their performance in both preclinical and clinical applications.

The review is organized as follows. The PAUS principle and signal processing are expressed in Section 2. The design of the PAUS probe and scanning mechanism of the PAUS system are summarized in Section 3 and Section 4, respectively. Section 5 presents the summary and future perspectives for the development of the PAUS probe and system.

## 2. PAUS Principle and Signal Processing

### 2.1. PAUS Principle

**Figure 1:**
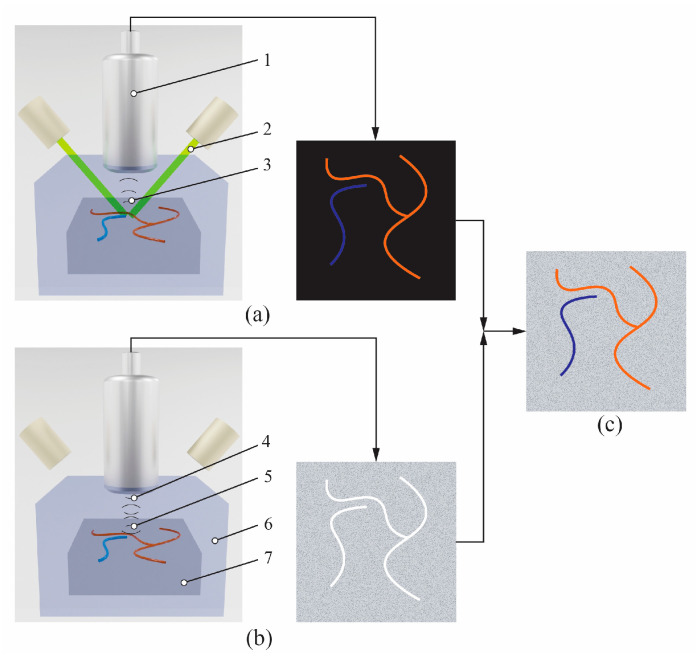
PAUS principle: (**a**) PA image, (**b**) US image, (**c**) overlaid PAUS image. 1—US transducer, 2—laser beam, 3—US waves generated by the PA effect, 4—US waves emitted from the transducer, 5—reflected US waves, 6—US propagation environment, 7—tissue sample.

PAUS imaging is a hybrid modality that integrates the principles of PA and US imaging to provide complementary functional and anatomical information of tissue. Figure 1 shows the PAUS principle. A laser beam generated by a laser source propagates to the tissue sample, where specific tissue components such as hemoglobin, melanin, lipids, and other chromophores absorb the light [50,80]. The absorbance process causes the temperature increase, which leads to thermoelastic expansion, resulting in US wave generation. A US transducer is used to collect these US waves to draw a PA image, as shown in Figure 1a. Based on the probe characteristics, such as transducer bandwidth, PA imaging offers high spatial resolution, which can illustrate the functional and molecular information [57,81].

Simultaneously, the same US transducer and pulsed radio frequency (P/R) are employed to emit and receive ultrasound waves that propagate through the tissue. According to the different properties of the sample structure, there are several US waves reflected off that are echo signals. The transducer captures these echo signals to generate a conventional US image, as shown in Figure 1b. The US image illustrates the structure information of the tissue based on the difference magnitude of reflected signals and the coherence summation during the image reconstruction [82,83]. During the scanning process, a coupling medium, such as US gel or deionized water, is employed between the transducer and tissue to reduce reflection, which enables better resolution of the resulting images [84].

The combined PA and US images are overlaid to produce the PAUS image, enabling co-registered visualization of optical absorption and acoustic properties, as shown in Figure 1c [85]. During the scanning process, both PA and US data are collected alternatively at each lateral position using the same data acquisition system (DAQ). This integrated approach allows for the concurrent visualization of vascular structures and tissue composition, providing both composition and structural insights [85,86].

### 2.2. PAUS Signal Processing

**Figure 2:**
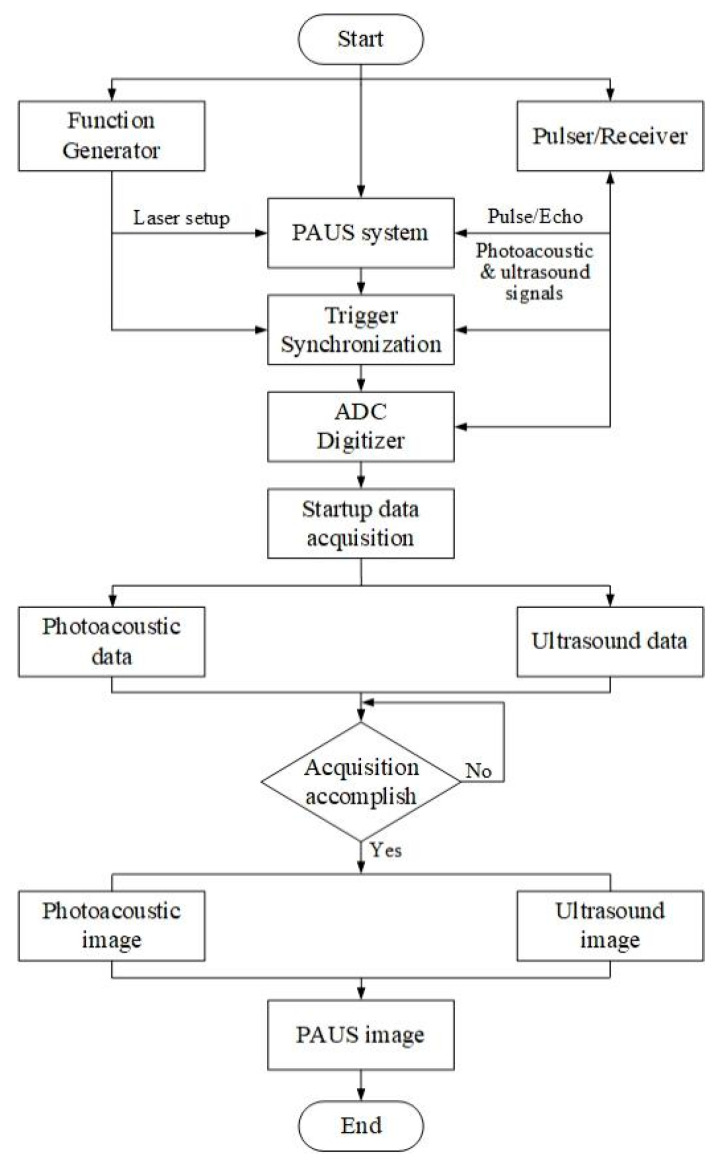
The flowchart of PAUS signal processing.

Figure 2 shows the flowchart of PAUS signal processing that uses a DAQ system to collect data and generate an image using image algorithm reconstruction. A function generator is used to create a trigger pulse for a short-pulsed laser that is connected to the PAUS probe and trigger synchronization. In US mode, a P/R is implemented to send an electric signal to the US transducer, thereby emitting US waves to the sample. Both PA and US signals are gathered by the same P/R that is connected to an analog–digital converter (ADC) digitizer. The trigger signals from the function generator and the P/R are synchronized with the encoder of the PAUS scanning system, allowing data acquisition at each lateral scanning position. Data collection is initiated simultaneously with probe scanning, and the PA and US signals are sequentially stored as separate datasets according to their time-of-flight responses until the acquisition is completed. The PA and US images are reconstructed using algorithms such as time domain, delay and sum, and time reversal [87,88,89,90]. Finally, the PAUS image is generated by co-registering and fusing the reconstructed PA and US images.

## 3. Design of PAUS Probe

According to the PAUS principle, a probe is designed by integrating optical and acoustic components to simultaneously acquire PA and US signals. Optical fibers and focusing lenses are employed to deliver pulsed laser illumination to the target tissue, while ultrasound (US) transducers and, in some designs, acoustic mirrors are used for signal detection and propagation [26,78,91,92,93,94,95,96,97,98,99,100,101,102,103,104,105]. These components are arranged and fixed within a dedicated housing, whose geometry and dimensions are primarily determined by the size and configuration of the US transducer. The US transducer can be either a single element [26,99,100,102,103,105] or an array transducer [78,91,92,93,94,95,97,98,101,104]. The locations of the lens and US transducer in the housing part can be adjusted to make the focal zone, thereby improving the resolution of the PAUS probe. Axial resolution is primarily determined by the US transducer’s bandwidth, while lateral resolution depends on the transducer receiver aperture [44,106,107,108]. Based on the data collection arrangement, it can be operated in either optical resolution (OR) or acoustic resolution (AR) modes. In OR mode, the excitation light was focused to define the lateral resolution. In contrast, AR mode relied on acoustic focusing, achieved through either focused or diffused optics, to determine the image resolution. The OR mode is only possible at a relative shallow depth, whereas the AR mode is appropriate for deeper penetration.

**Figure 3:**
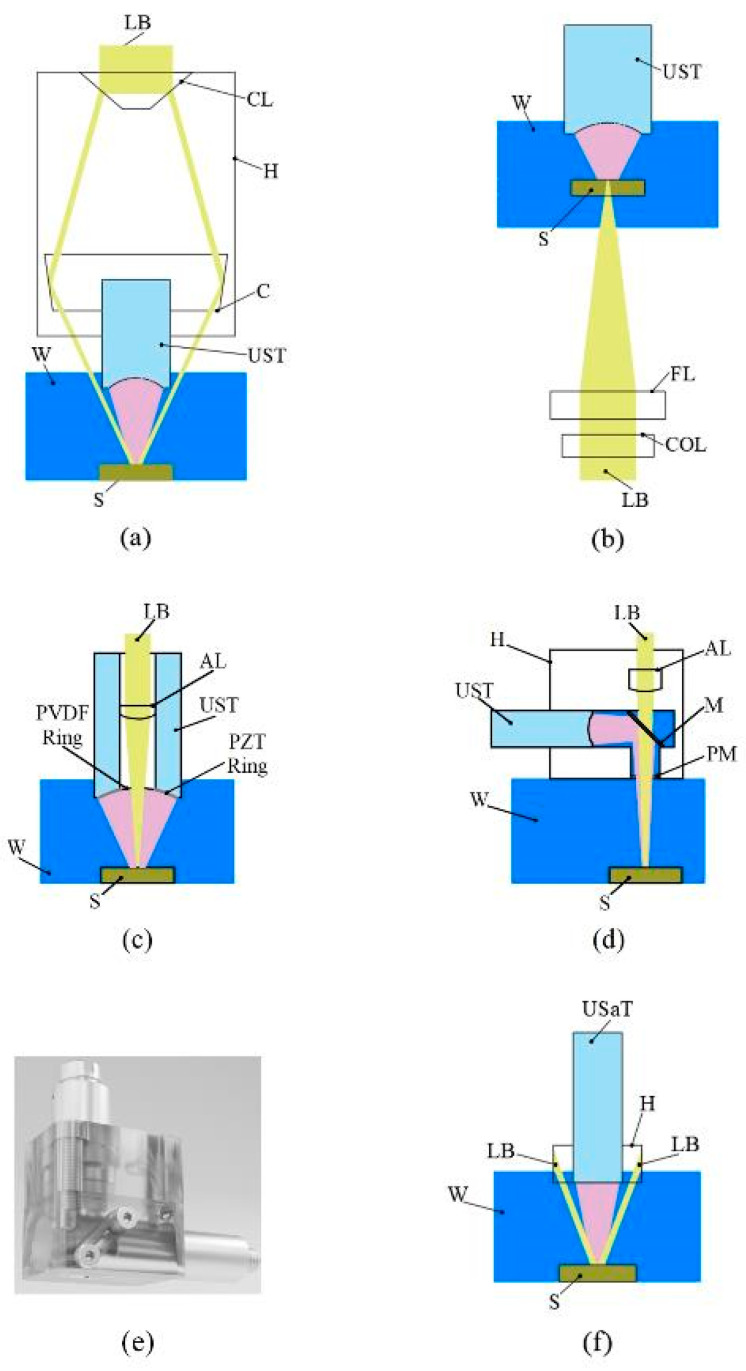
Schematic of PAUS probes: (**a**) reflection type, (**b**) transmission type, (**c**) customized US transducer type, (**d**) diagonal reflection type, (**e**) 3D-rendered design of diagonal reflection type, (**f**) US array transducer type. AL: aspheric lens, C: condenser, CL: conical lens, COL: correction lens, FL: focusing lens, H: housing part, LB: laser beam, M: mirror, PM: polyethylene membrane, S: sample, USaT: US array transducer, UST: US transducer, W: water.

The optical and acoustic components of a PAUS probe can be arranged on either the same side or opposite sides of the sample. Figure 3a shows a schematic of the PAUS probe in reflection type, in which both optical and acoustic components are located on the same side of the tissue. In this concept, the US transducer is put inside the optical condenser [105]. The condenser angle is calculated based on the measured focal length of the US transducer and the acoustic properties of the sample [109], while its size is determined by the transducer dimensions and the selected condenser angle. The housing part of this design is simple and practical for manufacturing and assembly. However, the probe is bulky because of the large size of the condenser component. In transmission type, the optical acoustic components are placed on the opposite side of the sample [92], as shown in Figure 3b. This concept uses the housing part without a condenser, thereby reducing the probe size, but it introduces challenges in achieving precise alignment between the laser beam and the US transducer.

To overcome the aforementioned limitations, two approaches are presented in published works. First, the US transducer is customized to incorporate optical components directly. A through-hole is created at the center of the transducer, allowing optical elements to be mounted within the backing layer [99]. Two coaxial rings of piezoelectric (PZT) and polyvinylidene fluoride (PVDF) materials are fixed on the external housing, as shown in Figure 3c. The PZT ring connects to a pulser/receiver (P/R) for US pulse emission, while the PVDF ring is used to detect both PA and US signals. This probe is very compact, with a length and diameter of 15 mm and 10 mm, respectively. Although this concept is small in size, modifying the transducer structure may adversely affect US performance. The second approach involves developing a housing part, where the laser beam incident and the US transducer are arranged in vertical and horizontal directions, respectively [26,110,111,112,113]. A diagonal prism or mirror slab is implemented in this concept to reflect the PA and US signals to the US transducer that is the diagonal reflection type. Figure 3d shows the schematic of this design, where the housing part is fabricated from acrylic material [26]. An open hole is created on bottom of the housing part to ensure that the laser beam propagates straight without diffraction. This hole is sealed with a polyethylene membrane to maintain the water in the US transducer chamber. The dimension of this probe is 40 mm × 20 mm × 45 mm, as shown in Figure 3e. This configuration simplifies focal alignment but requires high manufacturing precision, particularly in the placement of the focusing lens and US transducer.

In addition, the PAUS probe can be developed using a commercial US array transducer, which is designed for both hand-held and system scanning processes [94,98,114]. In these systems, the optical components are mounted on a housing part fixed to the US array transducer, as shown in Figure 3f. The US array transducers consist of 64 [95], 128 [96], or 256 [78,91] elements, which enable extending the scanning area. Recently, transparent ultrasound transducers have been developed to enable precise coalignment of acoustic and optical pathways while maintaining minimal acoustic coupling. This advancement facilitates real-time multimodal imaging of deep biological tissues [115]. Technically, the frequency of the US array transducer is low, thereby resulting in low resolution of the probe. For instance, the PAUS probe using a 128-element linear array transducer with a frequency of 10 MHz produces US lateral and axial resolutions of 600 μm and 400 μm, respectively [96]. When using a 25 MHz single-focused transducer, these resolutions are about 136 μm and 76 μm [26]. To enhance the spatial resolution of the PAUS probe based on an array transducer, the combination of PA with advanced US methods has been proposed, enabling super-resolution in the reconstructed images [116,117,118]. Furthermore, the image quality depends on the uniformity across various elements of the US array transducer, which leads to a reduction in image quality due to hardware limitations [119].

## 4. Design the Scanning Mechanism of the PAUS System

**Figure 4:**
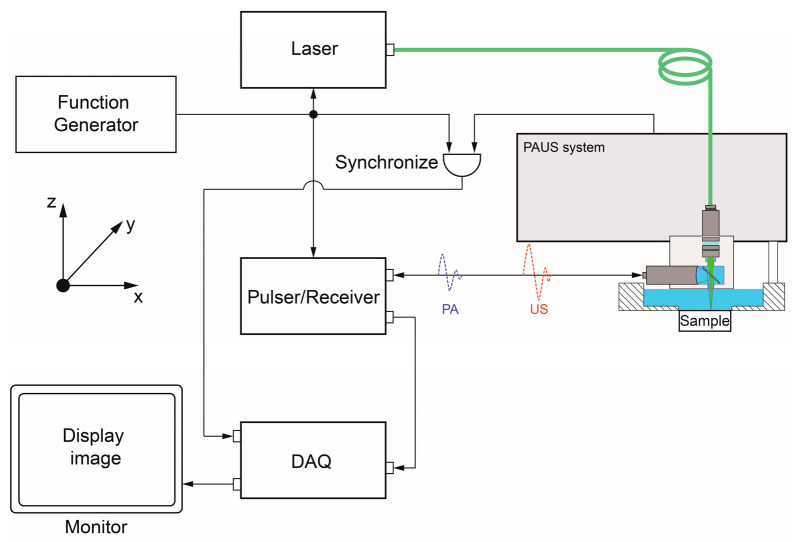
The schematic of the PAUS system.

The PAUS probe is attached to the scanning mechanism of the PAUS system that moves in sequence to draw the images. Figure 4 presents the schematic of the PAUS system. The signals of the laser function generator, P/R, and PAUS system are triggered simultaneously to record both PA and US data. Depending on the defined ROI, the PAUS system is configured and controlled to perform the scanning process following three steps. First, the PAUS probe and sample are arranged along the z-axis to align the focal zone. Second, the ROI parameters are specified to determine the data acquisition strategy. Third, the PAUS system conducts a motion to cover the whole ROI. Based on the PAUS probe, the motion of the PAUS system can be implemented in 1D or 2D directions. To conduct these movements, the scanning mechanism of the PAUS system is developed by exploiting the motion of some mechanisms, such as a motion stage, a voice coil, a combination of a slider crank and a ball screw, and a galvo.

### 4.1. PAUS System Motion Based on Motion Stage

**Figure 5:**
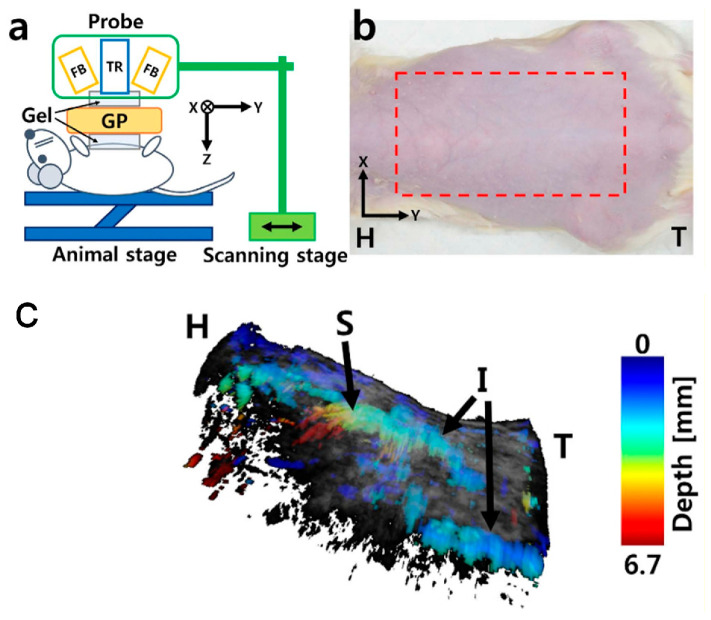
PAUS system using the motion stage: (**a**) schematic of the PAUS system, (**b**) photograph of the rat and ROI (the red dashed rectangle), (**c**) depth-resolved volumetric PAUS image of the rat. H: head, I: intestine, S: stomach, T: tail, FB: fiber bundle, TR: US array transducer, GP: gelatin pad [104].

**Figure 6:**
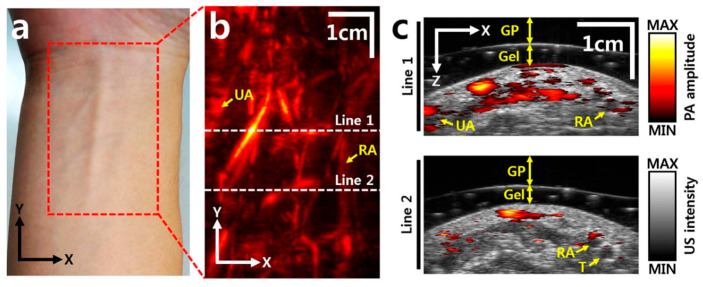
In vivo images of a human forearm: (**a**) photograph of a human right forearm, (**b**) PA image, (**c**) cross-sectional overlaid PAUS images at the two white dashed lines in (**b**). UA: ulnar artery, RA: radial artery, T: tendon, Gel: gelatin, GP: gelatin pad [104].

The motion stage is designed to meet the stringent requirements of high precision and compactness, both of which are particularly critical for integration into PAUS systems. The stage facilitates linear movement along one or multiple axes, driven by either traditional motors or brushless linear direct current motors (BLDCMs). The traditional motors can be stepper or servo and are connected to the ball screw mechanism to convert rotational motion to linear motion [101,104,105,120,121,122]. Due to the advantages of simplicity, low-cost, and versatility, this concept is commercialized by McMASTER-Carr [101], Velmex [122], and THORLABS [123]. Figure 5a shows the schematic of the PAUS system using a scanning stage and a probe with an array transducer. During the scanning process, this PAUS system moves in one direction to record a suitable ROI, as shown in Figure 5b. Figure 5c shows the depth-resolved volumetric PAUS image of the rat [104]. The ROI along the *x*-axis is covered by the PAUS probe equipped with a 128-element ultrasound array, while the scanning stage provides mechanical translation along the *y*-axis. Using this PAUS system, the human forearm is successfully captured in vivo, as shown in Figure 6. To achieve movement in two directions (*x*- and *y*-axes), the system can utilize either two ball screw mechanisms or a combination of a ball screw and a timing belt. Based on the features of the ball screw mechanism, the PAUS system can easily extend the ROI. However, the scanning speed of this design is relatively slow, which results in longer scanning times. For instance, to cover the scanning range of 75 mm, the scanning speed is 2.5 mm/s [104]. The scanning time is around 30 min to image an ROI of 60 mm × 40 mm [105].

Nowadays, to address the speed limitations, the motion stage is designed by exploiting the BLDCM. In contrast to traditional motor-driven systems, BLDCMs directly convert electrical energy into linear motion, thereby minimizing mechanical transmission components. Technically, this motor contains fixed permanent magnets and windings mounted on the guideway, enabling smooth, quiet, and high-speed motion based on electromagnetic actuation principles. High-resolution encoders are used to precisely control movement. THORLAB has developed a commercial type of this motion stage (model: M150XY/M) that provides 150 mm of travel in both the *x*- and *y*-axes at maximum speeds of 170 mm/s and 230 mm/s, respectively [124]. This type of motion stage provides significant advantages in terms of precision, speed, reliability, and reduced maintenance requirements; however, its major limitation remains the high cost, which is approximately USD 10,000 for the M150XY/M model.

### 4.2. PAUS System Motion Based on a Voice Coil Mechanism

The voice coil mechanism is a commonly used electromechanical device that operates based on the fundamental principles of electromagnetism. The key component is a wire cylinder (voice coil) that is assembled to a permanent magnet with a defined magnetic gap. When an electrical current passes through the coil, it generates a magnetic field that interacts with the static magnetic field of the permanent magnet, producing a force that drives the coil in a linear motion. Owing to its fast response, high precision, and friction-free operation, the voice coil actuator is particularly suitable for implementing fast-scanning modules in PAUS systems. In this concept, the PAUS probe is attached to the voice coil to enable rapid scanning along one axis, while the orthogonal axis is driven more slowly using a ball screw or timing belt mechanism.

**Figure 7:**
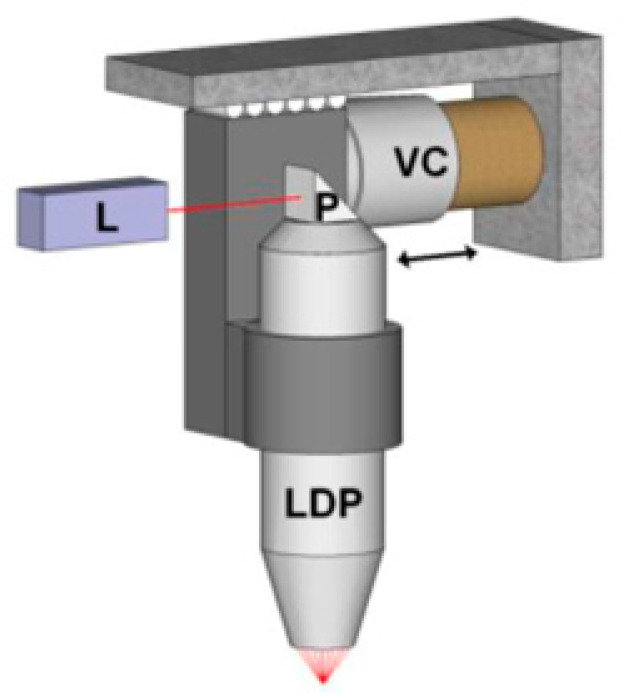
PAUS system using a voice coil mechanism. L: incident light from a laser, P: prism, VC: voice coil, LDP: light delivery probe. Reprinted with permission from [103]; © Optical Society of America.

**Figure 8:**
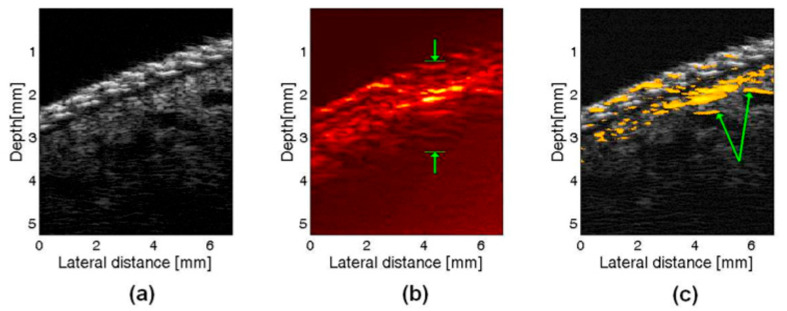
In vivo images of a a human finger: (**a**) US image, (**b**) PA image, (**c**) PAUS image, arrows indicate where large vessels are seen only in part in PA data. Reprinted with permission from [103]; © Optical Society of America.

Figure 7 shows the PAUS system using the voice coil stage (VSC-10-023-BS-01, from H2W Technologies, Inc., Valencia, CA, USA) that is driven by the motor controller (Elmo Harmonica HAR 5/60) [103]. The system achieves a scanning range of 9 mm at a frame rate of 20 fps, corresponding to an average scanning speed of approximately 1800 mm/s. The higher speed can be achieved by reducing the scanning ranges. Using the PAUS system with the voice coil stage, a human finger is successfully captured with in vivo imaging, as shown in Figure 8 [103].

The key advantages of the voice coil mechanism are simple and high accuracy because it operates without complex linkages and friction. However, expanding the scanning range and managing the vibration problem remain challenging. Key design parameters include the number of coil turns, coil geometry and material, force generation capability, and thermal performance. Increasing the coil length or diameter to expand the scanning range inevitably raises the coil mass, which can adversely affect force generation and dynamic response. During the scanning process, the coil undergoes a reciprocating motion, which produces a shaking force that causes the vibration problem. Consequently, the PAUS probe and coil are designed to minimize their masses to reduce the shaking force. Additionally, the heat dissipation requirements are essential for maintaining the system’s efficiency. Therefore, the overall system design requires a careful balance among thermal management, mechanical stability, and actuation performance through optimized material selection and dimensional design.

### 4.3. PAUS System Motion Based on a Combination of Slider Crank and Ball Screw Mechanisms

**Figure 9:**
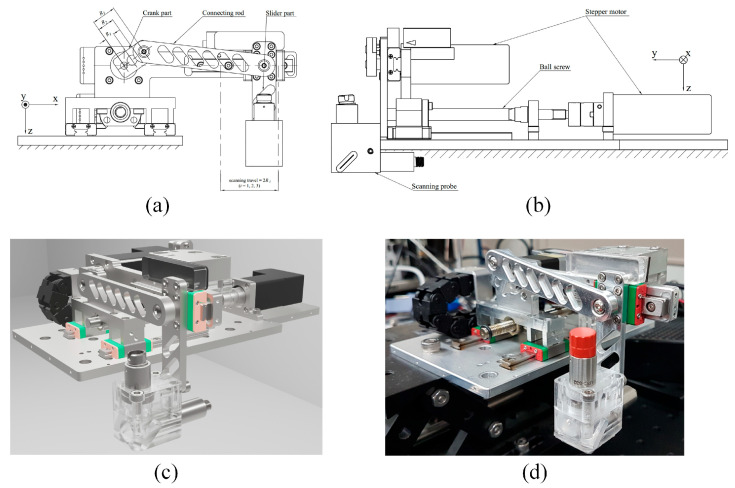
PAUS system using a combination of slider crank and ball screw mechanisms: (**a**) 2D front view, (**b**) 2D left side view, (**c**) 3D rendered design, (**d**) photograph of the entire system [26].

In this design, the PAUS system is developed by a combination of slider crank and ball screw mechanisms, which implement two linear motions along the *x*- and *y*-axes, respectively. By converting pure rotational motion from the crank part to pure linear motion of the slider part, the slider crank mechanism conducts a fast linear motion along the *x*-axis. Meanwhile, the ball screw mechanism provides a comparatively slow and precise linear motion along the *y*-axis for stepwise raster scanning. Figure 9 shows the PAUS system using a combination of slider crank and ball screw mechanisms. The slider crank mechanism consists of three main parts: a crank, a connecting rod, and a slider. The crank is directly coupled to the motor shaft to generate rotational motion, which is transmitted through the connecting rod to drive the linear motion of the slider. One complete revolution of the crank produces a full reciprocating cycle of the slider, corresponding to two linear strokes along the *x*-axis. The PAUS probe is attached to the slider part. The travel and velocity of the system along the *x*-axis are fixed by crank radius and crank rotational speed, respectively. Although this configuration offers a simple, high-speed, and cost-effective solution, it is limited to imaging a specific ROI. Adjusting the scanning characteristics for a different ROI requires redesigning the slider crank mechanism with new geometric parameters.

**Figure 10:**
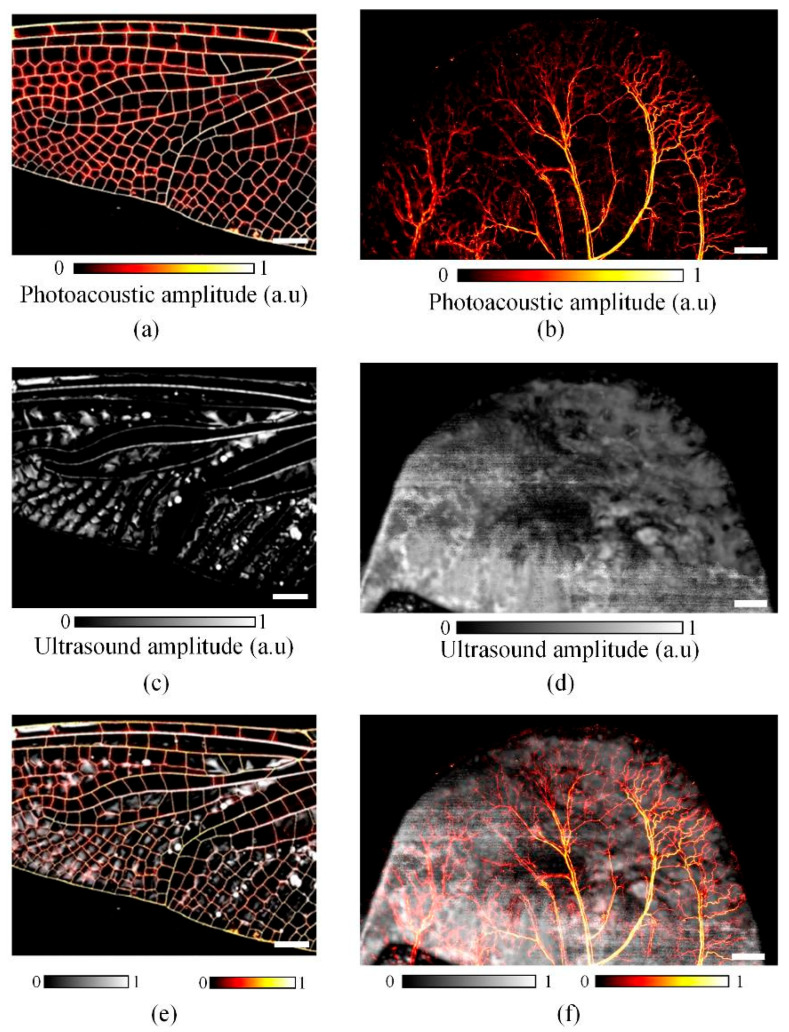
Dragonfly wing and mouse ear imaging: (**a**,**b**) PA images; (**c**,**d**) US images; (**e**,**f**) PAUS images [26]. The scale bar represents 2 mm.

Figure 10 presents the result images of dragonfly wings and mouse ear samples that are obtained using this PAUS system. The ROIs for the dragonfly wing and mouse ear were 18 mm × 14 mm and 26 mm × 16 mm, respectively. For the dragonfly wing, the crank radius and speed were set to 9 mm and 780 rpm (revolutions per minute), whereas for the larger mouse ear ROI, a crank radius of 13 mm and a rotational speed of 540 rpm were used. With a step size of 10 μm, the scanning times were 54 s and 89 s for acquiring the US images of the dragonfly wing and mouse ear, respectively [26]. Figure 10a,b show the PA images of the two samples, while Figure 10c,d illustrate the corresponding US images. The final PAUS images were generated by spatially co-registering and merging the PA and US images at each lateral scanning position, as shown in Figure 10e,f. Due to the inherent characteristics of the slider crank mechanism, increasing the crank radius to enlarge the ROI necessitates a reduction in rotational speed, which in turn decreases scanning speed and increases total acquisition time. To maintain a high scanning speed while expanding the ROI, two identical PAUS probes can be mounted on a single slider with a spacing equal to twice the crank radius [125]. Alternatively, a double slider crank mechanism has been introduced, in which two slider assemblies are arranged to achieve a total travel distance twice that of a conventional single mechanism [126]. In addition, high-speed operation may induce mechanical vibrations that degrade image quality. In that case, a counterweight is attached to the crank part to solve the vibration problem [127].

### 4.4. PAUS System Motion Based on the Galvo Mechanism

The galvo mechanism uses lightweight mirrors mounted on the galvanometers, which are controlled to rotate around a pivot point. The main components are the motor and detector. The mirror is affixed to the motor shaft, and its rotational motion is governed by electromagnetic principles. During its operation, the electric current passes through the coil, thereby generating the magnetic field. The rotation motion of the mirror is produced by the interaction between the magnetic field and the magnetic assembly. The detector is used to provide feedback on the mirror angular position, enabling closed-loop control for smooth and precise operation. By regulating the mirror motion, the galvo mechanism facilitates movement in 1D and 2D directions.

**Figure 11:**
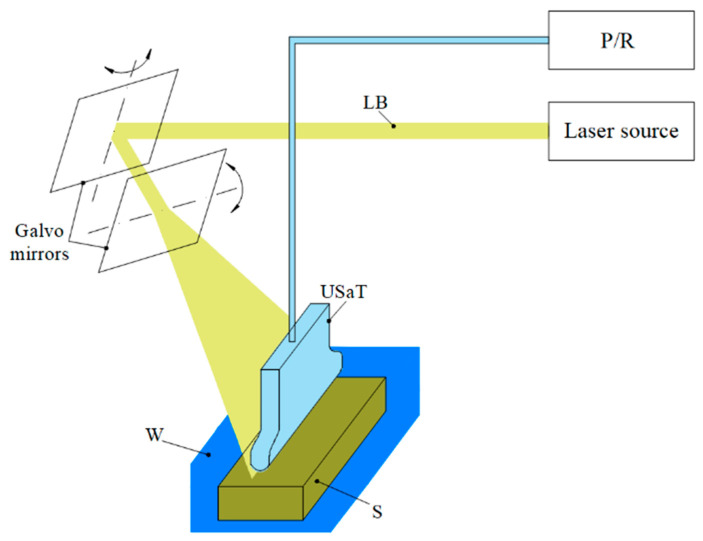
PAUS system using the galvo mechanism.

In the PAUS application, the galvo system is implemented to steer the laser beam [128,129]. Figure 11 shows the schematic of the PAUS system using the galvo mechanism. The laser beam is propagated to the mirror, which is adjusted through its angular movement to scan the ROI. In this version, the PAUS probe developed based on a US linear array transducer is used to receive the PA signal produced at a high frame rate. Using the galvo PAUS system, an ROI of 28 mm × 28 mm has been successfully imaged at 30 fps [128]. The advantages of the galvo mechanism include low inertial mass and the absence of mechanical contact between moving components, which make it particularly suitable for high-speed and high-precision imaging applications with enhanced durability and reliability. However, there are some limitations that need to be addressed in future studies. First, achievable ROI is relatively small and difficult to expand due to the limited angular range of the mirror. Second, the mirror steers the laser beam to various axial depths, resulting in misalignment between laser and ultrasound, which affects image quality. Third, the captured images must be reconstructed to correct the distortions caused by the scanning patterns, which necessitates the optimization of the image processing algorithm, particularly in real-time scanning process.

## 5. Summary and Future Perspectives

In this review, the design of dual-modal PAUS probes and systems is systematically presented and discussed. Numerous studies have demonstrated the potential of PAUS technology in both preclinical small-animal imaging and clinical human applications. This review aims to present the current development of PAUS technology in order to enhance its capabilities. Critical parameters such as scanning time, ROI, and image quality require continued improvement to support diagnostic decision-making. The fundamental principles of PAUS imaging and associated signal processing techniques are first introduced, as they form the basis for the design of PAUS probes and systems. The PAUS probe is developed using either a single-element or a linear array US transducer, each offering distinct spatial resolutions and thus influencing overall image quality. The characteristics of the existing PAUS probe are summarized in Table 1.

To extend the ROI and increase the scanning speed, the PAUS system is developed by leveraging the advantage motion of some mechanisms. Current systems support 1D and 2D movements enabled by motion stages, voice coils, slider crank and ball screw combinations, and galvo mechanisms. A comparative overview of the advantages and limitations of these PAUS system designs is provided in Table 2.

According to the summary of the PAUS probes and systems, several approaches for improvement should be addressed in future studies as follows:

(1) Improve image reconstruction tools to achieve uniform contrast across elements of linear array PAUS probes and synchronize signal-to-noise ratios of all elements to enhance overall image quality, which can be achieved by enabling the signal amplitude adjustment for each element of the linear array transducer.
(2) Optimize image processing algorithms to handle the distortion problems when using the linear array PAUS probe to record images in real-time, thereby facilitating its broader adoption in clinical diagnosis and interventional applications. The distortion problems can be solved by adding the artificial intelligence (AI) module to the image processing framework.
(3) Develop a linear array PAUS probe applied to the PAUS system utilizing a combination of slider crank and ball screw mechanisms to significantly reduce the scanning time that supports timely decision-making in both preclinical and clinical studies.
(4) Design a PAUS system capable of simultaneous three-axis motion to scan the 3D sample without requiring a tillable, such as the foot and forearms. These movements can be controlled by a combination of motions of some mechanisms, such as a ball screw, a timing belt, and a slider crank.
(5) Develop portable, compact, and high-performance PAUS systems suitable for widespread use in educational and scientific purposes by customizing the shape, size, and functional capabilities of all PAUS system components.
(6) Integrate PAUS systems with deep learning and artificial intelligence frameworks to significantly enhance image processing capabilities and expand diagnostic potential for human diseases.

## Figures and Tables

**Table 1:** Comparison of various PAUS probes.

Laser Source	Center Frequency of the US Transducer (MHz)	No. of Elements	Resolution (µm)			References	Advantages	Disadvantages
			PA Lateral	US Lateral	Axial			
Q-switched pump laser with a wavelength that ranges from 690 to 930 nm	5	Single	760	1160	310	[105]	- Simple design and practical for manufacturing and assembly - Deep penetration	- Low resolution - Large size of the probe - Only suitable for use with a scanning system
Q-switched diode-pumped solid-state laser with a wavelength of 532 nm and a PRR of 25 kHz	25	Single	10.2	136	76	[26]	- Compact size of the probe - High resolution	- Complex housing component - Shallow depth - Only suitable for use with a scanning system
A pulsed laser with a wavelength of 532 nm and a PRR of 10 kHz	35	Single	6.8	45	57.8	[99]	- Compact size of the probe - High resolution	- Modify the structure of the US transducer - Shallow depth - Only suitable for use with a scanning system
A Q-switched Nd:YAG laser with a wavelength of 1064 nm and a PRR of 20 kHz	10	128	693	600	400	[96]	- Simple design of the probe - Large scanning coverage of B-scan - Deep penetration - Appropriate for use with hand-held and scanning systems	- Low resolution - Distorted image - High cost
A pulsed Nd:YAG-pumped laser with a wavelength that ranges from 680 to 980 nm and a PRR of 25 kHz	7.5	256	870 ÷ 1440	930 ÷ 1430	90 ÷ 130	[91]	- Simple design of the probe - Large scanning coverage of B-scan - Deep penetration - Appropriate for use with hand-held and scanning systems	- Low resolution - Distorted image - High cost

**Table 2:** Comparison of various PAUS systems.

Motion Type	ROI	Scanning Speed	Step Size	Scanning Time	References	Advantages	Disadvantages
Motion stage	60 mm × 40 mm	-	0.2 mm and 0.5 mm in the *x*- and *y*-axes, respectively	30 min	[105]	- Simple design - Facilitate the extension of ROI - Appropriate for a probe with a single element and an array transducer	- Low speed - Long-time consumption
Voice coil	9 mm in one direction	20 fps	-	-	[103]	- Simple design - High speed - Short-time consumption - Appropriate for a probe with a single element and an array transducer	- Small scanning coverage - Vibration heat generation problems
A combination of slider crank and ball screw mechanisms	10 mm × 4 mm	30 fps	10 μm	14 s	[26]	- Simple design - High speed - Short-time consumption - Appropriate for a probe with a single element and an array transducer	- Fixed scanning coverage - Vibration problem
	18 mm× 14 mm	26 fps		54 s			
	26 mm × 16 mm	18 fps		89 s			
Galvo	28 mm × 28 mm	30 fps	-	-	[128]	- Simple design - High speed - Short-time consumption	- Only suitable for a probe with an array transducer - Low resolution - Distorted image

## Data Availability

No new data was created in this study.
